# Family socioeconomic status and moderate to vigorous physical activity among Chinese adults: the chain mediating roles of exercise environment and exercise motivation

**DOI:** 10.3389/fpubh.2026.1737196

**Published:** 2026-02-03

**Authors:** Yibo Gao, Mingzhe Li, Lupei Jiang, Xiang Pan, Yichuan Tian, Yanfeng Zhang, Koya Suzuki

**Affiliations:** 1 China Institute of Sport Science, Beijing, China; 2 Graduate School of Health and Sports Science, Juntendo University, Inzai, Japan; 3 Juntendo Administration for Sports, Health and Medical Sciences, Tokyo, Japan; 4 School of Politics and Public Administration, Soochow University, Suzhou, China; 5 Institute of Health and Sports Science & Medicine, Juntendo University, Inzai, Japan

**Keywords:** chain mediation, exercise environment, exercise motivation, family socioeconomic status, moderate to vigorous physical activity

## Abstract

**Objective:**

This study aimed to examine the relationship between family socioeconomic status (SES) and moderate to vigorous physical activity (MVPA) among adults aged 20–59 years, as well as the chain-mediating effects of exercise environment (EE) and exercise motivation (EM) in this association. Methods: Using data from the 2020 National Fitness Survey, a total of 55,804 adults aged 20–59 years were included in the analysis. Multiple linear regression, chain-mediation modeling, and subgroup analyses were performed using SPSS 30.0.

**Results:**

(1) Family SES was positively associated with MVPA (*r* = 0.053, *p* < 0.01). (2) EE and EM mediated the relationship between family SES and MVPA through three distinct pathways: the independent mediation of EE (effect size = 0.439, 95%CI = 0.387 ~ 0.494), the independent mediation of EM (effect size = 0.168, 95%CI = 0.141 ~ 0.195), and the chain mediation of EE and EM (effect size = 0.170, 95%CI = 0.128 ~ 0.151), accounting for 48.67, 18.63, and 15.52% of the total effect, respectively. (3) The direct effect of family SES on MVPA exhibited differential trends across age (20–29, *β* = 0.090, 95%CI = 1.139 ~ 1.726, *p* < 0.01, 30–39, *β* = 0.076, 95%CI = 0.854 ~ 1.311, *p* < 0.01, 40–49, *β* = 0.059, 95%CI = 0.731 ~ 1.282, *p* < 0.01, 50–59, *β* = 0.081, 95%CI = 1.410 ~ 2.101, *p* < 0.01), sex (male, *β* = 0.089, 95%CI = 1.315 ~ 1.709, *p* < 0.01, female, *β* = 0.014, 95%CI = 0.033 ~ 0.430, *p* < 0.05), and urban–rural subgroups(urban, *β* = 0.038, 95%CI = 0.469 ~ 0.814, *p* < 0.01, rural, *β* = 0.043, 95%CI = 0.592 ~ 1.160, *p* < 0.01), with significant interaction effects observed.

**Conclusion:**

This study reveals that family SES influences MVPA among adults through three pathways: EE alone, EM alone, and the chain-mediation of EE and EM. These findings suggest potential entry points for strengthening family level support for physical activity and may inform strategies aimed at improving adults’ exercise participation.

## 1 Introduction

The health benefits of sufficient physical activity (PA) are beyond dispute, yet nearly one-third (31%) of adults worldwide still fail to meet recommended PA levels (1). Physical inactivity is a well-established behavioral risk factor associated with increased morbidity and premature mortality (2). According to the World Health Organization, adults aged 18–64 years should accumulate at least 150–300 min of moderate-intensity aerobic activity, 75–150 min of vigorous-intensity activity, or an equivalent combination per week (3). Physical inactivity is estimated to reduce global life expectancy by approximately 0.68 years based on modeling studies that assume all adults achieve recommended activity levels (4).

For adults, the family is a primary life domain that shapes daily routines and health-related behaviors (5). Family socioeconomic status (SES) reflects educational, occupational, and economic resources that shape daily living conditions, lifestyle patterns, and health beliefs, which may influence participation in moderate to vigorous physical activity (MVPA) (6). Even in adulthood, family SES continues to shape daily activity opportunities. In this study, SES was conceptualized at the family level because income, educational attainment, and occupational status collectively reflect the socioeconomic resources available within the adult’s living environment and remains a key determinant of neighborhood choice and access to exercise opportunities among adults. Thus, family SES provides a broader structural indicator than individual SES alone.

Exercise environment (EE) refers to the accessibility of sport and physical activity facilities in the spaces where individuals live (7). Research has shown that proximity to exercise facilities, especially within walking distance, is a key component of an activity supportive environment (8). Greater facility availability provides more opportunities for convenient participation, reduces time and access barriers, and is positively associated with higher levels of physical activity (9). Consistent evidence indicates that adults who live near a greater variety of nearby sport or recreational facilities tend to accumulate more MVPA (10). In the context of socioeconomic disparities, higher Family SES is more likely to reside in neighbourhoods with better access to such facilities, which may partially explain family SES differences in physical activity (2, 11).

Exercise motivation (EM) is often conceptualized within the self-determination theory (SDT), which distinguishes between intrinsic motivation driven by enjoyment or personal value and extrinsic motivation driven by external rewards or pressures (12, 13). Higher levels of self-determined motivation are associated with greater persistence and higher PA levels (14). In the present study, exercise motivation reflects the degree to which individuals value, enjoy, or feel competent in PA (15). Research indicates that EM is likewise positively linked to MVPA (16). Highly motivated individuals more readily translate activity intentions into next-day MVPA (17). Prior studies indicate that exercise motivation is associated with higher levels of MVPA and may act as a psychological mechanism linking family SES to activity behavior (18). Higher family SES may provide greater access to resources and opportunities that support the development of stronger exercise motivation.

Moreover, EE may influence motivation by reducing participation barriers and providing supportive conditions for regular activity (19). Individuals living in higher quality environments may experience greater convenience and perceived support, which are associated with stronger motivation and increased engagement in MVPA. Therefore, exercise environment and exercise motivation may function sequentially in the association between socioeconomic status and MVPA.

**Figure 1:**
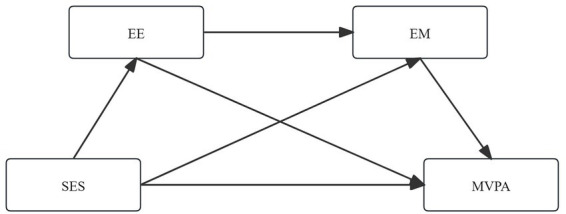
Relationship between SES, EE, EM, and MVPA. SES, family socioeconomic status; EE, exercise environment; EM, exercise motivation; MVPA, moderate to vigorous physical activity.

Physical activity behavior in China is also shaped by cultural and structural contexts, including marked urban–rural differences in activity norms and facility availability (20). Moreover, data collection overlapped with the early phase of the COVID-19 pandemic, during which changes in mobility patterns and perceptions of public spaces may have influenced activity opportunities (21). These contextual factors warrant consideration when interpreting SES-related disparities. Although previous studies have demonstrated the importance of family SES, EE and EM in explaining MVPA among adults, existing studies still have limitations. Most studies have examined only a single mediating mechanism and lacked systematic validation of the interaction between EE and EM. Furthermore, research on adult populations remains relatively scarce, with insufficient large-scale national data analysis. Based on the above evidence, this study proposes the following hypotheses. Hypothesis 1: Higher socioeconomic status is associated with higher levels of MVPA. Hypothesis 2: The exercise environment mediates the association between socioeconomic status and MVPA. Hypothesis 3: Exercise motivation mediates the association between socioeconomic status and MVPA. Hypothesis 4: The exercise environment and exercise motivation function sequentially in the association between socioeconomic status and MVPA. Figure 1 presents the hypothesized serial mediation model, illustrating the proposed pathways linking family SES to MVPA through EE and EM.

Aims of the present study are to: (1) quantify the total effect of family SES on adults’ MVPA; (2) evaluate the independent mediating roles of EE and EM in the SES-MVPA pathway; and (3) test the serial EE-EM mediation effect. By examining the pathways linking family SES to MVPA, this study aims to provide evidence that may support the development of targeted strategies to promote MVPA among adults.

## 2 Methodology

### 2.1 Participants

The data for this study were derived from the 2020 National Fitness Survey (22), which covered 31 provinces (autonomous regions and municipalities) across China and included survey responses from adults aged 20 to 59 years. The survey was conducted from January 1 to March 31, 2020. A three-stage probability proportional to size (PPS) sampling method was employed. In the first stage, a total of 471 county-level units were selected across the 31 provinces, with the number of sampled counties in each province determined by provincial population size. In the second stage, 13 village or neighbourhood committees were randomly selected within each sampled county, resulting in 5760 sampled village units. In the third stage, 12 adults aged 20 to 59 years were randomly selected from each village or neighbourhood committee using local household registration lists. A total of 59,747 samples were collected.

### 2.2 Variables

#### 2.2.1 Family socioeconomic status

Family SES was calculated based on income, education level, and occupational type (23). Income was classified into eight categories, education into five levels, and occupation into five types. Prior to principal component analysis (PCA), the following steps were taken: (1) The assigned values for each indicator were standardized using Z-scores, and the correlations among the standardized indicators were examined. The Kaiser–Meyer–Olkin (KMO) measure of sampling adequacy was 0.651, and Bartlett’s test of sphericity was significant (χ^2^ = 105,013.392, *p* < 0.001), confirming the suitability of the data for PCA. (2) One principal component with an eigenvalue greater than 1 (eigenvalue = 1.802) was extracted, accounting for 63.077% of the total variance. Although we considered the possibility of a multi-factor structure, the scree plot demonstrated a clear “elbow” after the first component, and no other components possessed an eigenvalue greater than 1.0, supporting a uni-dimensional solution. The factor loadings for income, education level, and occupational type were 0.735, 0.780, and 0.808, respectively. To further validate the convergence, we examined the communalities, which were 0.540, 0.608, and 0.653, respectively. All values exceeded the recommended threshold of 0.5, indicating that the single component adequately captured the variance of the underlying indicators. Thus, family SES was computed as: family SES = (0.735 × Z_income_ + 0.780 × Z_education_ + 0.808 × Z_occupation_)/1.802. Higher scores indicate higher family SES. This composite indicator reflects broader household level socioeconomic resources rather than individual attributes, and such PCA based SES indices are widely applied in population-based studies (24, 25). In this study, education level refers to the respondent’s own educational attainment rather than parental education.

#### 2.2.2 Exercise environment

EE was measured by assessing the availability of physical activity facilities within a 15 min walking distance from the participants’ residences. Participants were asked: “Which of the following types of sports venues or facilities are located within a 15 min walk from your home?” The question listed 23 common types of sports facilities (e.g., football fields, basketball courts, table tennis venues) and allowed respondents to add any other relevant facilities not listed. Each selected facility type was assigned 1 point, and the total score was calculated. This unweighted facility count approach is widely used in built environment and physical activity research to represent overall opportunity for activity, rather than the specific function or scale of each facility (9, 11). This measure reflects the accessibility based definition described in the introduction, where proximity and variety of nearby facilities represent key elements of an activity supportive environment. Higher scores indicate a more favorable exercise environment.

#### 2.2.3 Exercise motivation

EM was assessed using the Chinese version of the Measurement of Physical Activity Motivation—Revised (MAPM-R) scale. This instrument assesses motivation across five dimensions: enjoyment, competence, appearance, health, and social interaction. It consists of 15 items rated on a 5-point Likert scale, with higher scores indicating stronger motivation. Although grounded in the broader of SDT, the MPAM-R captures multiple self-determined components, particularly enjoyment and competence, which align with the motivational processes described in the introduction. In this study, the total score was used to represent overall motivation strength. The MAPM-R has been translated and validated in a Chinese adult population (26), with an overall Cronbach’s *α* of 0.737 and subscale α coefficients ranging from 0.725 to 0.899. In the present study, the scale demonstrated good reliability, with an overall Cronbach’s α of 0.790 and subscale α coefficients ranging from 0.756 to 0.826.

#### 2.2.4 Moderate to vigorous physical activity

PA levels were assessed using the International Physical Activity Questionnaire Short Form (IPAQ-SF), which collects self-reported data on participants’ PA over the past week. The IPAQ-SF has been validated in Chinese adults, with intraclass correlation coefficients (ICCs) of 0.79 (0.66–0.88) for total PA, 0.85 (0.75–0.91) for moderate PA, and 0.75 (0.60–0.85) for vigorous PA (27).

#### 2.2.5 Control variables

Based on previous research (28), the following demographic variables were included as controls: (1) gender (1 = male, 2 = female), (2) age (continuous variable), and (3) residence (1 = urban, 2 = rural).

### 2.3 Survey

Prior to data collection, a standardized training session was conducted for technical supervisors from all 31 provinces (autonomous regions and municipalities). These supervisors then provided secondary training to the interviewers in their respective regions to ensure a consistent understanding of the survey protocol and research objectives, thereby minimizing interviewer bias. During the formal survey, participants were sampled based on information provided by local statistical bureaus. Face-to-face interviews were conducted in households. Before each interview, the purpose and significance of the study were explained to the participants, and written informed consent was obtained. To protect privacy, all personal identifiers were anonymized using a three-level coding system. The study was approved by the Ethics Committee of the China Institute of Sport Science (Approval No. CISSLA-20191029).

### 2.4 Statistical analysis

Normality of variables was assessed using the Kolmogorov–Smirnov test. Continuous variables are presented as mean ± standard deviation (X ± SD), and categorical variables as frequencies and percentages (n, %). Sample weights were calculated and applied based on the age-specific and urban–rural distribution data of the 20–59 years old population from the 2020 Seventh National Population Census to enhance the representativeness of the sample. Group differences were examined using one-way ANOVA or Chi-square test. Harman’s single-factor test was used to check for common method bias. Pearson correlation analysis was used to examine bivariate relationships, and multiple linear regression was employed to analyze influencing factors. Model assumptions including linearity, normality of residuals, and homoscedasticity were examined and met. Mediation effects were tested using the SPSS macro-PROCESS 4.0 (29) with bias-corrected bootstrapping (5,000 resamples). A 95% confidence interval not including zero indicated a significant mediation effect. Subgroup regression analyses were conducted by gender, residence, and age group to explore potential differences in the direct effect of family SES on MVPA. For all regression analyses, 95 percent confidence intervals were reported and used together with *p*-values to assess the statistical significance of effects. All analyses were performed using SPSS version 30.0 (IBM Corp, Armonk, NY, USA), with a *p*-value < 0.05 considered statistically significant.

## 3 Results

After data cleaning and exclusion of invalid responses, a total of 55, 804 adults were included in the final analysis, corresponding to an effective response rate of 93.4%. Common method bias was assessed using Harman’s single-factor test. The results revealed three factors with eigenvalues greater than 1. The first factor accounted for 31.57% of the variance, which is below the critical threshold of 40%, indicating that common method bias was not a significant concern in this study. Therefore, the data were deemed suitable for subsequent mediation effect analysis (30). A multivariate linear regression analysis was conducted, with MVPA acting as the dependent variable. The variance inflation factors (VIFs) ranged from 1.008 to 1.265 and were all significantly below 10 (31), indicating that there were no issues of multicollinearity within the model. The model demonstrated an overall satisfactory fit, consistent with the assumptions of multivariate regression analysis.

### 3.1 Descriptive statistics

The final analytical sample consisted of 55,804 valid respondents. Among them, 28,427 (50.9%) were male and 27,377 (49.1%) were female. Regarding residence, 36,256 (65.0%) lived in urban areas and 19,548 (35.0%) in rural areas. Significant differences were observed across age groups for all variables except gender (all *p* < 0.001) (Table 1).

**Table 1:** Demographics.

Variables	20–29 Years	30–39 Years	40–49 Years	50–59 Years	20–59 Years	Statistical	*P*
Gender, *n* (%)						4.65	0.199
Male	5,697 (51.1)	7,420 (50.2)	7,640 (51.1)	7,670 (51.4)	28,427 (50.9)		
Female	5,462 (48.9)	7,356 (49.8)	7,311 (48.9)	7,248 (48.6)	27,377 (49.1)		
Residence, *n* (%)						160.75	<0.001
Urban	6,766 (60.6)	9,801 (66.3)	10,131 (67.8)	9,558 (64.1)	36,256 (65.0)		
Rural	4,393 (39.4)	4,975 (33.7)	4,820 (32.2)	5,360 (35.9)	19,548 (35.0)		
SES	3.75 ± 1.35	3.73 ± 1.47	3.34 ± 1.43	2.95 ± 1.30	3.42 ± 1.43	146.69	<0.001
EE	2.90 ± 1.72	2.95 ± 1.83	2.73 ± 1.82	2.37 ± 1.62	2.73 ± 1.76	129.88	<0.001
EM	3.53 ± 0.46	3.53 ± 0.46	3.51 ± 0.47	3.52 ± 0.47	3.52 ± 0.47	18.63	<0.001
MVPA/(Min·week^−1^)	122.52 ± 109.62	119.77 ± 106.46	137.69 ± 111.79	153.66 ± 136.86	134.18 ± 109.14	122.30	<0.001
Total, *n* (%)	11,159 (20.0)	14,776 (26.5)	14,951 (26.8)	14,918 (26.7)	55,804		

Occupation 1 represents temporary workers, unemployed individuals, or unskilled laborers; Occupation 2 denotes manual workers and self-employed persons; Occupation 3 refers to general management personnel as well as general professional and technical staff; Occupation 4 indicates middle management and mid-level professional and technical staff; and Occupation 5 corresponds to senior managers and senior professional and technical staff. The chi-square test was used to analyze categorical variables including gender and residence, the statistical measure is χ^2^. One-way ANOVA was employed to examine differences in SES, EE, EM and MVPA, the statistical measure is F.

### 3.2 Correlation analysis

Correlation analyses (Table 2) showed that family SES was positively correlated with EE (*r* = 0.314, *p* < 0.01), EM (*r* = 0.104, *p* < 0.01), and MVPA (*r* = 0.053, *p* < 0.01), indicating that adults with higher family SES reported better exercise environments, stronger motivation, and slightly higher MVPA. EE was positively correlated with both EM (*r* = 0.168, *p* < 0.01) and MVPA (*r* = 0.115, *p* < 0.01). A positive correlation was also found between EM and MVPA (*r* = 0.190, *p* < 0.01), suggesting that individuals with stronger motivation tend to engage in higher MVPA. With the exception of the non-significant correlation between EM and Residence, all four main variables were significantly correlated with the control variables (*p* < 0.01). Age was positively correlated with MVPA (*r* = 0.076, *p* < 0.01), while all other correlations between the main variables and control variables were negative (*p* < 0.01).

**Table 2:** Correlation between variables.

Variables	Age	Gender	Residence	SES	EE	EM	MVPA
Age	1						
Gender	−0.006	1					
Residence	−0.024**	−0.033**	1				
SES	−0.223**	−0.054**	−0.306**	1			
EE	−0.077**	−0.007	−0.200**	0.314**	1		
EM	−0.031**	−0.047**	−0.008	0.104**	0.168**	1	
MVPA	0.076**	−0.021**	−0.051**	0.053**	0.115**	0.190**	1

**p < 0.01; *p < 0.05.

### 3.3 Mediation effect analysis

A series of multiple linear regression models using a stepwise approach were conducted to examine the relationships between family SES (independent variable), MVPA (dependent variable), EE and EM (mediating variables), while controlling for gender, age, and residence. To indicate model explanatory power, *R*^2^ values for model 1 ~ 4 ranged from 0.009 ~ 0.052 (Table 3). In Model 1, age was positively associated with MVPA (*β* = 0.074, 95%CI = 0.146 ~ 0.182, *p* < 0.01), whereas gender and residence (gender, *β* = −0.023, 95%CI = −1.491 ~ −0.691, *p* < 0.01, residence, *β* = −0.050, 95%CI = −2.964 ~ −2.126, *p* < 0.01) showed negative associations. In Model 2, after adding family SES, age remained positively associated with MVPA (*β* = 0.089, 95%CI = *p* < 0.01), while gender and residence (gender, *β* = −0.018, 95%CI = −1.291 ~ −0.491, *p* < 0.01, residence, *β* = −0.031, 95%CI = −1.988 ~ −1.104, *p* < 0.01) remained negatively associated. Family SES was positively associated with MVPA (*β* = 0.063, 95%CI = 0.910 ~ 1.213, *p* < 0.01), indicating that higher family SES was associated with higher MVPA levels. In Model 3, after further adding EM, the relationships for the control variables remained consistent. Both family SES (*β* = 0.033, 95%CI = 0.405 ~ 0.717, *p* < 0.01) and EM (*β* = 0.108, 95%CI = 0.871 ~ 1.023, *p* < 0.01) were positively associated with MVPA, suggesting that adults with stronger motivation accumulated more MVPA. In Model 4, after adding EE, the control variables again showed consistent relationships. Family SES (*β* = 0.022, 95%CI = 0.215 ~ 0.522, *p* < 0.01), EE (*β* = 0.081, 95%CI = 0.631 ~ 0.783, *p* < 0.01), and EM (*β* = 0.176, 95%CI = 0.576 ~ 0.633, *p* < 0.01) were all positively associated with MVPA, showing that better facility availability and higher motivation each contributed to higher MVPA.

**Table 3:** Regression results of the chain mediation model.

Variables	Model 1				Model 2				Model 3				Model 4			
	*b*	SE	*β*	95%CI	*b*	SE	*β*	95%CI	*b*	SE	*β*	95%CI	*b*	SE	*β*	95%CI
Age	0.164	0.009	0.074**	0.146 ~ 0.182	0.196	0.010	0.089**	0.177 ~ 0.215	0.201	0.010	0.091**	0.182 ~ 0.219	0.202	0.009	0.092**	0.184 ~ 0.221
Gender	−1.091	0.204	−0.023**	−1,491 ~ −0.691	−0.891	0.204	−0.018**	−1.291 ~ −0.491	−0.909	0.203	−0.019**	−1.307 ~ −0.511	−0.559	0.2	−0.012**	−0.951 ~ −0.167
Residence	−2.545	0.214	−0.05**	−2.964 ~ −2.126	−1.546	0.225	−0.031**	−1.988 ~ −1.104	−0.909	0.226	−0.018**	−1.351 ~ −0.467	−1.277	0.222	−0.025**	−1.712 ~ −0.841
SES					1.061	0.077	0.063**	0.910 ~ 1.213	0.561	0.079	0.033**	0.405 ~ 0.717	0.368	0.078	0.022**	0.215 ~ 0.522
EE									0.947	0.039	0.108**	0.871 ~ 1.023	0.707	0.039	0.081**	0.631 ~ 0.783
EM													0.605	0.014	0.176**	0.576 ~ 0.633
*F*	162.742				169.713				256.863				531.818			
*R* ^2^	0.009				0.012				0.022				0.052			

*b*, unstandardized coefficient; SE, standard error; *β*, standardized regression coefficient; **p < 0.01; *p < 0.05.

**Figure 2:**
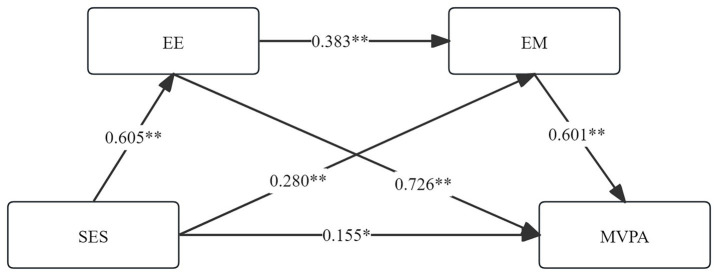
Chain mediation effect. SES, family socioeconomic status; EE, exercise environment; EM, exercise motivation; MVPA, moderate to vigorous physical activity; the coefficients in the figure are standardized coefficients. **p < 0.01; *p < 0.05.

Mediation analysis was performed using the SPSS macro-PROCESS 4.0. The results (Table 4) indicated significant indirect effects of SES on MVPA, as the bias-corrected confidence intervals for all paths did not include zero. This confirms the significant mediating roles of the two proposed mediators. The total indirect effect was comprised of three specific paths: (1) SES → EE → MVPA, with an effect value of 0.439 (95% CI = 0.387 ~ 0.494); (2) SES → EM → MVPA, with an effect value of 0.168 (95% CI = 0.141 ~ 0.195); and (3) SES → EE → EM → MVPA, with an effect value of 0.170 (95% CI = 0.128 ~ 0.151). All three pathways indicated positive associations, meaning that higher SES was linked to better EE, stronger EM, and subsequently higher MVPA. The contribution rates of these three paths were 48.67, 18.63, and 15.52%, respectively (Table 4; Figure 2).

**Table 4:** Standardized direct and indirect pathways.

Pathways	**Effect**	**Boot SE**	**95%CI**		**Contribution rate (%)**
			**LLCI**	**ULCI**	
Total effect	0.902	0.072	0.761	1.042	100.00
Direct effect	0.155	0.074	0.010	0.230	17.18
Total indirect effect	0.747	0.030	0.688	0.807	82.82
SES-EE-MVPA	0.439	0.027	0.387	0.494	48.67
SES-EM-MVPA	0.168	0.014	0.141	0.195	18.63
SES-EE-EM-MVPA	0.170	0.006	0.128	0.151	15.52

5000 Bootstrap samples.

To further explore the relationship between family SES and MVPA, stratified regression analyses were conducted by age group, gender, and residence, adjusting for confounding factors (Table 5). The results showed that family SES was significantly and positively associated with MVPA across all age groups. The strongest association was observed in the 20–29 age group (*β* = 0.090, 95%CI = 1.139 ~ 1.726, *p* < 0.01), followed by the 50–59 group (*β* = 0.081, 95%CI = 1.410 ~ 2.101, *p* < 0.01), the 30–39 group (*β* = 0.076, 95%CI = 0.854 ~ 1.311, *p* < 0.01), and the 40–49 group (*β* = 0.059, 95%CI = 0.731 ~ 1.282, *p* < 0.01). In the gender stratification, the association between family SES and MVPA was more pronounced in males (*β* = 0.089, 95%CI = 1.315 ~ 1.709, *p* < 0.01) compared to females (*β* = 0.014, 95%CI = 0.033 ~ 0.430, *p* < 0.05). Regarding residence, SES was positively associated with MVPA in both urban (*β* = 0.038, 95%CI = 0.469 ~ 0.814, *p* < 0.01) and rural (*β* = 0.043, 95%CI = 0.592 ~ 1.160, *p* < 0.01) populations. These findings suggest that the promotive effect of family SES on physical activity is not homogeneous but is moderated by age, gender, and place of residence. These findings suggest that the association between family SES and MVPA varies by age, gender, and residence.

**Table 5:** Stratified analysis.

Groups	*b*	SE	*β*	95%CI
20–29 Years	1.433	0.150	0.090**	1.139 ~ 1.726
30–39 Years	1.083	0.117	0.076**	0.854 ~ 1.311
40–49 Years	1.007	0.140	0.059**	0.731 ~ 1.282
50–59 Years	1.755	0.176	0.081**	1.410 ~ 2.101
Male	1.512	0.101	0.089**	1.315 ~ 1.709
Female	0.232	0.101	0.014*	0.033 ~ 0.430
Urban	0.642	0.088	0.038**	0.469 ~ 0.814
Rural	0.876	0.145	0.043**	0.529 ~ 1.160

*b*, Unstandardized coefficient; SE, standard error; *β*, standardized regression coefficient; **p < 0.01; *p < 0.05.

## 4 Discussion

This study examined how family SES shapes adults’ MVPA through a Chain mediation pathway involving EE and EM (SES → EE → EM → MVPA). Using a nationwide sample across 31 provinces, this study systematically integrated family SES, EE, and EM to test both independent and Chain mediation effects, addressing gaps in previous research that focused on single mediators or limited populations. Our findings provide novel evidence on the mechanisms linking socioeconomic status to physical activity, offering actionable insights for interventions and policies to enhance adult MVPA and promote health equity.

### 4.1 Direct effect of family socioeconomic status on moderate to vigorous physical activity

In this study, family SES demonstrated a significant positive association with MVPA (accounting for 82.82% of the total effect). This pattern may reflect several interrelated mechanisms. Adults with higher family SES generally possess higher levels of education, income, and occupational prestige (32). (1) Economic advantages can provide greater access to activity-supportive environments and opportunities, which may facilitate stronger autonomous motivation and lower participation barriers. Adults with higher family SES also tend to have better access to health information and engage more in health-promoting behaviors, which are associated with higher MVPA levels. Households with higher family SES often reside in neighborhoods with more accessible exercise facilities (33), a factor shown to support greater physical activity participation (34). Conversely, residents with lower family SES may face constraints that limit their use of available facilities (35). (2) Higher educational levels may enhance individuals’ understanding of health-related information (36) and support competence related beliefs that facilitate the internalization of MVPA values, consistent with SDT (37). This may help individuals appreciate the long-term value of MVPA and support more stable patterns of participation. These psychosocial mechanisms are associated with more consistent engagement in MVPA (38). (3) Higher occupational level may also be linked to broader social capital and health-supportive norms (39), which can influence adults’ opportunities and readiness to engage in MVPA. Adults with higher family SES may also have more stable careers and greater flexibility in leisure time allocation, which has been associated with higher MVPA participation (40). Overall, these factors interact through improved health literacy, access to supportive environments and motivational processes, contributing to higher MVPA.

The interaction effects in this study revealed a non-linear trend where the influence of family SES on MVPA first weakened and then strengthened with age. During young and middle adulthood, increased work and caregiving demands may limit discretionary time for PA. Even with high family SES, its translation into physical activity behavior is hindered, thus weakening its positive effect (41). From age 50 onwards, as family responsibilities diminish and health risks become more apparent, individuals with high family SES are more likely to translate health cognition into active behavior, thereby strengthening the influence of family SES (42). The SES-MVPA association was stronger among men, who may have more leisure time available for structured PA (43). The association was also stronger in rural areas, where more accessible outdoor spaces and lower participation costs may support PA participation (44).

### 4.2 Mediating effect of exercise environment

This study found that family SES was positively associated with EE among Chinese adults, a result consistent with findings from other countries (7). Adults with higher family SES often reside in neighbourhoods with greater access to sports and recreational resources. Higher family SES groups may also have greater access to health information and resources that support perceived competence and autonomy, thereby facilitating more autonomous motivation under SDT (45). In contrast, adults with lower family SES may experience greater barriers to accessing health information and PA opportunities, which can make it more challenging to develop autonomous motivation and consistent activity habits. These differences may contribute to the socioeconomic gradients observed in motivation and MVPA (46). Furthermore, this study also confirmed a positive correlation between EE and adult MVPA, consistent with prior research showing that proximity and accessibility of facilities support regular physical activity (47). Supportive physical environments can foster more enjoyable and competence enhancing experiences, which are fundamental to autonomous motivation in SDT (14). When adults perceive their environment as safe, convenient, and supportive, they are more likely to engage in positive activity experiences (48). Positive experiences within supportive environments may reinforce perceived competence and enjoyment, further strengthening autonomous motivation (49). This process may support more stable and sustained engagement in PA. Particularly in the context of the widespread sedentary lifestyle in contemporary China (50), supporting autonomous motivation remains an important psychological factor associated with adults’ willingness to engage in physical activity.

### 4.3 Mediating effect of exercise motivation

This study found that family SES was positively associated with EM among Chinese adults, consistent with results from other countries (51, 52). Adults with higher family SES often have greater access to health information and activity supportive environments, which may help foster stronger autonomous forms of motivation. In the SDT, autonomous forms of motivation such as engaging in MVPA because it is enjoyable or personally meaningful reflects higher perceived autonomy and competence. EM was also positively associated with adult MVPA in this study, consistent with evidence that individuals who experience great enjoyment and value in MVPA are more likely to maintain regular participation (53). These findings suggest that family SES difference in access to supportive conditions may contribute to variation in autonomous motivation, which is in turn associated with MVPA.

### 4.4 Chain mediating effect of EE and EM

The chain mediation model constructed in this study (family SES → EE → EM → MVPA) illustrates the roles of EE and EM in the association between family SES and adult MVPA. Specifically, EE represents an environmental pathway through which socioeconomic resources may relate to variation in MVPA. Supportive environments may foster greater perceived autonomy, competence, and enjoyment, which are central components of autonomous motivation in SDT. These motivational processes are in turn associated with higher MVPA. This suggests that family SES may relate to physical activity both through access to supportive environments and through psychological mechanisms linked to autonomous motivation. As mentioned, higher family SES is often associated with more supportive physical environments (18). Research indicates that characteristics such as accessibility, convenience, and perceived safety can support more positive activity experiences, which are linked to higher motivation (54). From an SDT perspective, environments that support autonomy and competence can facilitate the internalization of motivation, which is associated with more consistent MVPA participation (55). Supportive social environments, such as positive neighborhoods interactions and available community resources, have also been associated with higher perceived support for physical activity, which may relate to stronger motivation (56–58). Adults in such environments may find it easier to engage in activity that aligns with their motivational preferences, thereby supporting more regular participation. These combined processes are associated with more regular participation in physical activity.

These findings offer practical directions for MVPA promotion. Improving access to safe and affordable facilities may help reduce family SES-related disparities in MVPA, and combining such environmental improvements with strategies that strengthen autonomous motivation—especially among young adults—may enhance effectiveness. The mediation proportions observed here are broadly consistent with prior work showing that environmental and motivational pathways explain part of socioeconomic differences in PA (11, 14, 18). From a socio-ecological and self-determination theory perspective, these results suggest that interventions should address both opportunity and motivation. Future studies using longitudinal or objective PA measures are needed to verify these pathways.

### 4.5 Limitations

This study has several limitations. First, MVPA and EM were measured using self-reported questionnaires, which may introduce recall and social desirability biases. Second, the cross-sectional design precludes any causal inference, and potential endogeneity cannot be ruled out. Third, the sample included only adults aged 20–59 years, which limits the generalisability of the findings to youth and older adults. Fourth, although Harman’s test indicated no substantial method bias, this approach is limited in its ability to detect subtle forms of bias. Finally, the survey was conducted before the COVID-19 pandemic, and subsequent shifts in mobility patterns and use of public spaces may affect the applicability of the findings to current conditions.

## 5 Conclusion

This study identified a sequential pathway through which both the exercise environment and exercise motivation help explain socioeconomic differences in adults’ MVPA. The strength of these pathways varied across demographic groups, with the clearest family SES gradients observed among younger adults. These findings highlight the importance of simultaneously addressing environmental opportunities and motivation-related processes when promoting physical activity in populations facing socioeconomic disadvantage. The results also reinforce socio-ecological and motivational perspectives on health behavior, suggesting that interventions that combine accessible facilities with autonomy-supportive strategies may have potential to promote MVPA, particularly in groups facing socioeconomic disadvantage. Future longitudinal or experimental research is needed to assess the stability of these pathways and to test combined EE–EM interventions in low-SES populations.

## Data Availability

The data analyzed in this study is subject to the following licenses/restrictions: data are available upon reasonable request to the corresponding author. Requests to access these datasets should be directed to Yanfeng Zhang. zhangyanfeng0310@126.com.
